# Cognitive-motor dual-task training differentially affects visual working memory, multifocal attention, and multisensory integration in youth soccer players

**DOI:** 10.3389/fspor.2026.1863511

**Published:** 2026-07-22

**Authors:** Szabolcs Székely, Anna Gergely, János Horváth, József Topál

**Affiliations:** 1 Sports Cognitive Science Research Group, Institute of Cognitive Neuroscience and Psychology, HUN-REN Research Centre for Natural Sciences, Budapest, Hungary; 2 Adaptation Research Group, Institute of Psychology, Eötvös Loránd University, Budapest, Hungary; 3 Lendület Psychobiology Research Group, Institute of Cognitive Neuroscience and Psychology, HUN-REN Research Centre for Natural Sciences, Budapest, Hungary; 4 Experimental Psychology Research Group, Institute of Cognitive Neuroscience and Psychology, HUN-REN Research Centre for Natural Sciences, Budapest, Hungary; 5 Institute of Psychology, Károli Gáspár University of the Reformed Church in Hungary, Budapest, Hungary

**Keywords:** cognitive-motor dual-training, development, multifocal attention, multisensory integration, soccer, working memory

## Abstract

Cognitive and physical efforts draw on shared resources that are essential for athletic performance. However, traditional laboratory-based interventions often lack ecological validity and show limited transfer to real-game contexts. Cognitive-motor dual-task (CMDT) training addresses this limitation by integrating cognitively demanding tasks into sport-specific motor practice. This study examined the effects of a 10-month CMDT intervention on visuospatial working memory, multifocal attention, and multisensory integration. The sample comprised 34 youth soccer players (mean age = 9.2 ± 1.82 years) recruited from a talent development center. Participants were evenly allocated to either a CMDT intervention or a control group based on their biological and sporting age as well as their coaches’ evaluation of player ability. Cognitive performance was assessed using three screen-based tasks – Visuospatial Working Memory (VSWM), Multiple Object Tracking (MOT) and Sound-Induced Flash Illusion (SIFI) – to evaluate pre- to post-intervention changes in both groups. While no significant CMDT-related improvement was observed in spatial transformation and perspective-taking abilities (VSWM task), the primary analyses suggested CMDT-related improvements in multifocal attention (MOT task), and more robust effects were observed for multisensory integration (SIFI task). These findings suggest that embedding cognitive demands into sport-specific motor practice may be an effective approach to strengthening executive functions that are essential for high-level athletic performance.

## 1 Introduction

Cognitive and physical efforts are closely coupled, because both rely on partly overlapping attentional and executive resources and can therefore influence each other's performance (1, 2). This coupling is particularly relevant in team sports, where athletes must anticipate and respond to teammates and opponents while executing complex motor actions and making decisions under time pressure (3, 4). High-level performance therefore depends not only on automatized motor patterns, but also on the ability to flexibly adapt actions to rapidly changing environmental demands (5).

These considerations have motivated growing interest in the relationship between cognitive functions and sports performance. However, this field faces a significant methodological challenge: cognitive processes can be trained and measured under highly controlled laboratory conditions, but such conditions cannot adequately mirror the demands of real-life situations (6), which unfold under physical and emotional stress, strict time constraints, and complex perception-action demands. Because skill generalization is typically weak across domains or situations that are only loosely related to one another (7), screen-based executive-function training may improve performance on trained or closely related laboratory tasks, but evidence for transfer to real sport performance remains inconsistent (8). Thus, the limited far-transfer of laboratory-based cognitive gains remains a fundamental challenge for coaching in team sports.

Recent work on athletic skill development therefore emphasizes how cognitive and motor components are combined during practice. Traditional training methods often separate perceptual-motor components, such as technical skills, from perceptual-cognitive components, such as tactical decision-making (9), whereas training appears to be more effective when cognitive tasks are directly related to the motor component (10). This supports the idea that technical and tactical skills should be acquired simultaneously, within the same context and in an integrated manner (11).

This methodological approach is referred to as *cognitive-motor dual-task* (CMDT) training. Originally developed in rehabilitation contexts (12), CMDT training has increasingly been adopted in sports science. Its core principle is to combine cognitively demanding tasks with sport-specific motor actions, thereby approximating the demands of actual competition more closely than isolated cognitive training (13). CMDT has been shown to be effective across several team sports, including basketball (14) and rugby (15). This training approach may be particularly beneficial for younger athletes, as it can support the development of both motor skills and cognitive functions (16). Consistent with this view, Casella and colleagues (17) reported that a 10-week CMDT intervention improved visual search abilities and cognitive flexibility in 10-year-old soccer players, while van der Niet and colleagues (18) found that cognitively demanding aerobic exercise enhanced visual working memory in children of a similar age.

Building on these findings, the present study examined the potential effects of a 10-month CMDT intervention on three cognitive abilities considered theoretically relevant to soccer performance. The intervention was designed to increase the cognitive demands of regular soccer exercises by requiring players to perceive, maintain, update, and respond to spatially distributed information while moving. Accordingly, we selected three complementary outcome measures expected to be sensitive to such training demands: visuospatial working memory, multifocal attention, and multisensory integration.

*Visuospatial working memory (VSWM)* was selected because it supports visual search by guiding gaze allocation (19) and mediating selective attention throughout the search process (20). In soccer, VSWM may help players maintain and update information about player positions, movement directions, and spatial relations while adapting their actions to changing game conditions. Supporting this link, Mahfoudh and Zoudji (21) showed that expert soccer players outperform novices in visuospatial recall of dynamic, soccer-specific scenes, likely reflecting more efficient extraction and maintenance of relevant visual information. In the context of CMDT, VSWM is therefore relevant because young players must retain spatially distributed information while moving and translate it into ongoing motor responses. This is especially important in the U8–U12 age range, as school age represents a sensitive period for the integrated development of motor and cognitive functions. Recent evidence indicates that coordinative motor interventions can improve VSWM performance in primary school children (22), suggesting that this ability may be sensitive to cognitively demanding movement-based training during this developmental period.

It is also widely accepted that typical competition situations in team sports place extreme demands on *multifocal attention* – the capacity of the neural system to track several elements at once. This is because successful decision-making in such contexts often requires enhanced visual processing, including the ability to monitor multiple players, objects or events simultaneously. Liu et al. (23) found that both sport expertise and sport involvement were associated with better MOT performance, and increasing attentional load appears to impair intermediate athletes and non-athletes more strongly than elite players (24). However, it remains unclear, whether the superior attentional capabilities reflect innate predispositions or the effects of prolonged, high-level training (25). In the present study, the MOT task was therefore selected not simply as a general measure of attention, but as an experimental index of dynamic visual tracking under cognitive load. This is particularly relevant for soccer, where players must continuously monitor multiple moving elements, such as teammates, opponents, and the ball, while updating their action plans in response to rapidly changing spatial relations. Although, to our knowledge, no study has directly validated the specific MOT parameters used here against soccer-specific attentional load, the task parameters were chosen to approximate core attentional operations relevant to open-skill team sports: object speed increases temporal pressure, the number of targets and distractors manipulates multifocal attentional load, and the trial structure requires continuous updating of target locations once targets become visually indistinguishable from distractors.

The third cognitive process targeted in the present study was multisensory integration, referring to neural mechanisms that synthesize cross-modal, particularly visual and auditory, information (26). Multisensory integration plays a fundamental role in motor skill acquisition (27), and a recent model of elite soccer performance emphasizes that efficient perception-action coupling depends on the accurate processing of both external sensory cues and internal bodily signals (28). In dynamic game situations, players must integrate visual information about player movement and ball trajectory with auditory cues from teammates, coaches, referees, ball contact, and environmental events. The Sound-Induced Flash Illusion (SIFI) is a widely used paradigm for assessing audiovisual temporal integration (29). It measures susceptibility to audiovisual illusions in which auditory tones alter the perceived number of visual flashes, producing either fission or fusion percepts. Although multisensory processing may play an important role in team sport performance, empirical evidence on the effects of perceptual training on athletes’ audiovisual timing precision remains limited [but see (30) for findings from a non-athlete sample]. Therefore, SIFI was included not as a direct measure of soccer-specific performance, but as an exploratory mechanistic index of audiovisual temporal integration. This rationale is supported by evidence suggesting that player position may influence audiovisual integration in soccer, with goalkeepers exhibiting a narrower temporal binding window than outfield players (31), although such differences may reflect enhanced unisensory precision rather than superior multisensory processing *per se* (32).

**Figure 1:**
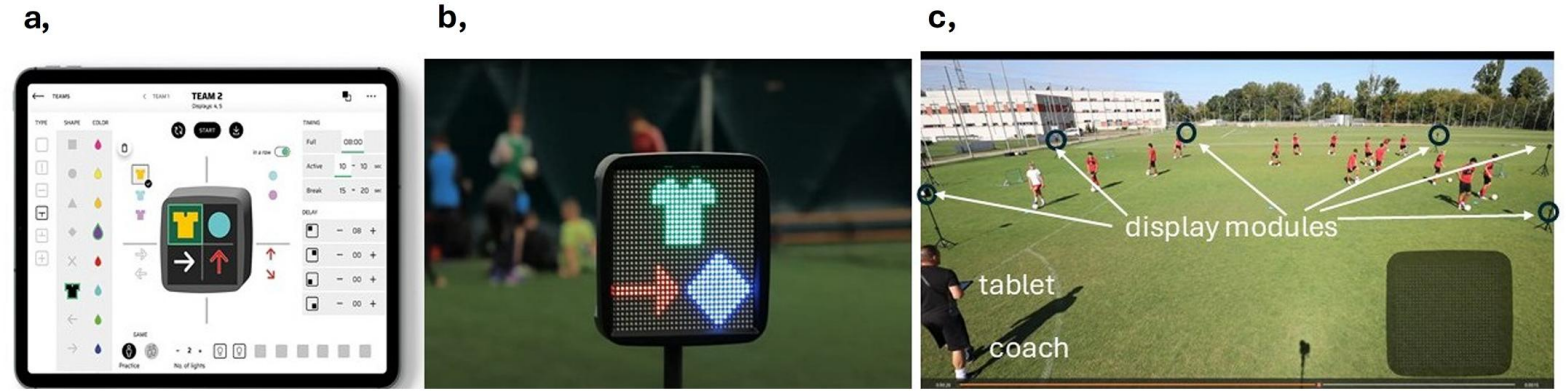
The NORT Complex smart coaching system. **(a)** WiFi-controlled interface (tablet operated by the coach); **(b)** programmable display module presenting visual task cues; **(c)** arrangement of display modules around the pitch during training.

To assess the effects of the CMDT intervention on visuospatial working memory, multifocal attention and multisensory integration, we used a newly developed smart coaching system, the NORT Complex^1^. It consists of eight programmable display modules positioned around the pitch, which deliver visual cues via a WiFi-controlled interface (Figure 1). These signals provide players with task-specific instructions that must be integrated into their ongoing movement patterns. This setup enables simultaneous engagement of movement coordination skills and key cognitive processes such as attentional control, perceptual processing, and working memory. Importantly, the key difference between traditional and NORT Complex training programs lies not in the content of the fieldwork – since both approaches focus on practicing fundamentally the same technical and tactical skills – but in the manner in which the coach delivers task-specific information to the players. Specifically, by replacing verbal instruction with visual information delivered via displays positioned at various locations in space, to which players must respond rapidly and in accordance with task requirements, an additional demand is placed on the athletes’ executive functions, thereby making the training exercises cognitively more demanding.

The aim of the present study was to examine whether 10 months of weekly CMDT training using the NORT Complex smart coaching system produces measurable effects on cognitive functions considered relevant to youth soccer performance. We predicted that, compared with traditional training, the CMDT intervention would affect U8–U12 soccer players’ visuospatial working memory, multifocal attention, and multisensory integration.

## 2 Methods

### 2.1 Participants

A total of 34 male, school-age registered soccer players representing different age divisions (U12: *n* = 2; U11: *n* = 10; U9: *n* = 10; U8: *n* = 4; U7: *n* = 10; mean age ± SD = 9.2 ± 1.82 years) were recruited from a talent center in March 2024. All participants had been certified athletes for at least one year and, as such regularly underwent sports medical examinations; none of them had any diagnosed visual or hearing impairments. Participants were paired based on three criteria: biological age, sporting age (i.e., the number of semesters they had been playing soccer) and their coaches’ evaluation of player ability, rated on a 7-point scale. Within each pair, players were then assigned to either the CMDT intervention or the control group by the technical manager of the talent center, who was not aware of the potential effects of CMDT training or the specific hypotheses of the present study (Table 1). The study was conducted in accordance with the guidelines of the American Psychological Association and was approved by the United Ethical Review Committee for Research in Psychology (EPKEB 2024/48). Children's caregivers provided written informed consent prior to participation.

**Table 1:** Demographic characteristics of the CMDT and control groups as the time of the children's assignment (March 2024).

Descriptive statistics						
	Age (months)		Sporting age (semester)		Rating of player ability	
	Control	CMDT	Control	CMDT	Control	CMDT
Mean	109.5	111.3	7.5	8.1	4.1	4.1
SD	22.8	21.6	3.4	3.2	0.9	0.9
Range	73.0–144.0	67.0–145.0	2–13	3–14	3–5	3–5

### 2.2 Procedure

The procedure consisted of three phases:

- Pre-intervention measurements of visual working memory, multifocal attention and multisensory integration using screen-based tasks (April-May 2024) – see below for detailed descriptions of the cognitive task protocols.
- The intervention period (May 2024-March 2025) included all participants engaging in their regular training regimen, consisting of 3–5 sessions per week, except during the summer (6 weeks) and winter (3.5 weeks) off-seasons, as well as matches organized by their club. Children in the control group participated only in traditional training sessions (60 min. per session; mean number of sessions ± SD = 114.5 ± 17.3) whereas children in the CMDT intervention group took part in both traditional training sessions (60 min. per session; mean number of sessions ± SD = 81.7 ± 17.2) and cognitive-motor dual training using the NORT Complex smart coaching system (60 min. per session; mean number of CMDT sessions ± SD = 49 ± 4.9). Based on these attendance records, the estimated structured training exposure was 114.5 h in the control group and 130.7 h in the CMDT intervention group. Thus, the CMDT group received approximately 14.1% greater total training exposure than the control group. However, the distribution of training types differed between groups: the control group completed more traditional soccer training sessions, whereas the CMDT group completed fewer traditional sessions but additionally participated in CMDT sessions delivered through the NORT Complex system.
- Post-intervention recordings of the same screen-based tasks used in phase I.

#### 2.2.1 Task protocols

Children were tested individually in a distraction-free room at the training centre. Upon arrival, participants were seated in front of a laptop (Dell Latitude E7450 or an ASUS Zenbook 14OLED). Before beginning the task, the experimenter provided detailed information about the sequence and nature of each task to help ensure that participants felt comfortable during the experimental procedure. Each participant completed the three tasks in the same order: first the Visuospatial Working Memory (VSWM) task, second the Multiple Object Tracking (MOT) task and finally the Sound-Induced Flash Illusion (SIFI) task.

##### 2.2.1.1 Visuospatial working memory task (VSWM)

Based on findings that VSWM performance is closely related to object-based rotation capacities and perspective-taking [i.e., participant-based rotation; see (33)], we developed a task in which participants were required to observe and memorize spatial arrangements of objects from a sideline perspective and then identify the same arrangement from options presented from a top-down perspective. The task consisted of two training and eighteen test trials. During the introductory (training) phase of the task, participants were first presented with a blue blank screen for 5,000 ms, followed by the presentations of a table evenly divided into an 8 × 8 grid of identical fields, with two black and two white pawns arranged from a sideline perspective. After viewing and memorizing the ’sample layout’ for 5,000 ms, four possible options were displayed on the screen from a top-down perspective. Participants were instructed to select the option they believed matched the previously observed layout and to make their decision as quickly and accurately as possible. This instruction was intended to simulate an important aspect of real-life, sport-specific situations in which athletes must balance decision-making accuracy and reaction speed. The four options remained on the screen until the participant made a choice by pointing to one of them.

**Figure 2:**
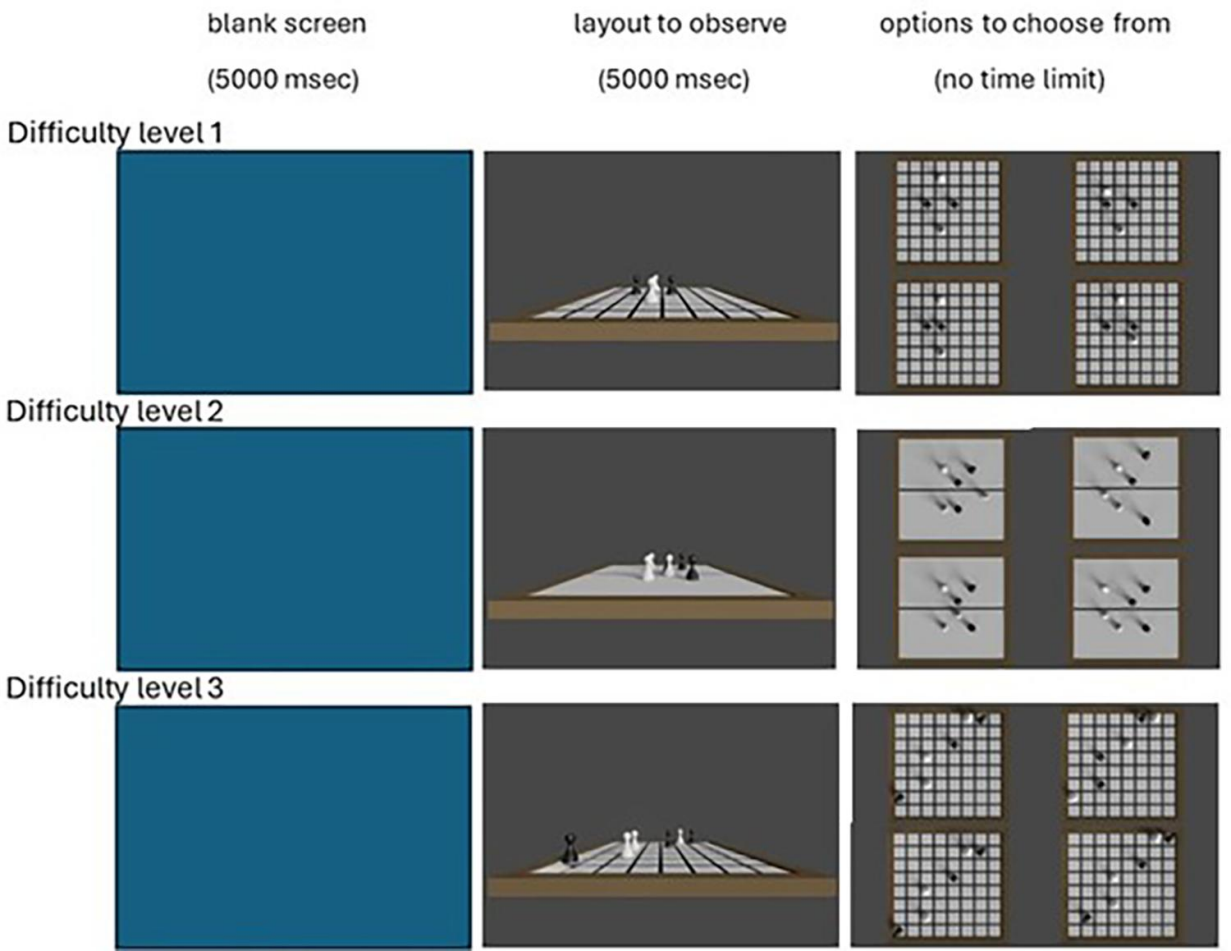
Examples of trials representing different difficulty levels in the VSWM task. Participants viewed a layout for five seconds and were then presented with four response options with no time limit. After their response and a five-second inter-trial interval, a new layout was presented. Rows illustrate increasing task difficulty, with higher levels requiring more precise encoding and transformation of the spatial arrangement.

Each trial was followed by a 5,000 ms inter-trial interval, during which a blue blank screen was displayed. It is important to note, that in the training trials the ’sample layout’ formed an easily recognizable shape (e.g., a rhombus – see Figure 2) and only one of the four options depicted that specific form. Thus, the participant's task was simply to recognize the shape of the ’sample layout’ rather than to memorize the detailed spatial arrangement of the items. After completing the two training trials, participants were asked to confirm their understanding and then completed a total of eighteen test trials. Each trial was video-recorded for later analysis of participants’ pointing responses.

The test trials represented three levels of difficulty (see Figure 2). Trials 1–8 corresponded to the same level of difficulty as the two training trials (i.e., an 8 × 8 - square grid with 4–6 black and white pawns, where only one of the four options depicted the shape presented in the sample layout – *difficulty level 1*). In the following four test trials (trials 9–12), the table was divided only by a halfway line, which made it somewhat more difficult to identify the option that matched the sample layout (*difficulty level 2*). The difficulty increased further in the final six test trials (trials 13–18), as the four alternative layouts in these trials represented the same shape as the sample layout. Consequently, participants had to observe and memorize the detailed spatial arrangement of the pawns in order to select the correct option (*difficulty level 3*). The task was administered during both the pre- intervention and post-intervention phases (mean ± SD time elapsed between the two recordings: 325 ± 14.9 days).

##### 2.2.1.2 Multiple object tracking task (MOT)

The MOT task has a substantial methodological literature [for a recent review, see (34)]. In the present study, the task was implemented in accordance with established protocols, without introducing methodological deviations or novel variations. A fixation cross appeared on the centre of the screen for 500 ms, followed by eleven randomly positioned circles: three green, three blue, two half green and half blue, and three white. Participants were instructed to memorize and track all circles that initially contained the color green – that is, the three fully green, and the two half-green circles. After a 4,000 ms flickering phase, all circles turned white and began moving randomly across the screen for 6,000 ms at varying speeds. When the movement phase ended, participants were asked to select the circles they believed had initially contained the color green by clicking on them with a mouse. Once selections were made, the next trial began after a 2000ms inter-trial interval. Participants completed five practice trials, which served multiple purposes. These trials ensured that the children understood the task and allowed the experimenter to assess whether they could effectively use the computer mouse to indicate their answers. If a child reported difficulty using the mouse, or if the experimenter observed significant problems, the experimenter himself recorded the child's responses instead, based on the child pointing to the chosen options with their finger. In total, 3 children completed the task in this manner. The training phase was then followed by fifteen test trials during the pre-intervention measurement and twenty trials during the post-intervention measurement (mean ± SD time elapsed between the two recordings: 351 ± 8.7 days).

##### 2.2.1.3 Sound induced flash illusion task (SIFI)

Participants were instructed to look at a grey plastic box (8 × 5 × 4.5 cm) with a 5 mm diameter multi-color light emitting diode (LED, WP154A4SUREQBFZGC, Kingbright Electronic Europe GmbH, Issum, Germany), on its front side. The plastic box was positioned at eye level, 30 cm to the left side of the laptop screen. The originally crystal-clear LED was diffused by sandpaper. Visual stimuli were 17-ms-long flashes of the blue (470 nm wavelength) LED channel with 25% pulse-width modulation (at 4,482 Hz). In each trial, one or two 17 ms flashes were presented. To ensure similar luminance conditions for flash perception, illumination in the experimental room was kept relatively constant (280–320 lux, measured by a Voltcraft LX-10, lux meter, Conrad Electronic SE, Hirschau, Germany) across participants and sessions. Children were asked to indicate the number of flashes they perceived by pressing the ‘1’ or ‘2’ key on the keyboard. The auditory stimuli were 3.5 kHz pure tones of 17 ms duration (including 2 ms linear rise and 2 ms linear fall times, 16 bit range, 44.1 kHz sampling rate, generated by Csound software^2^ version 8.16, presented through HD-25 headphones (Sennheiser, Wedemark, Germany) at 69 dB(SPL) intensity (measured by a HSUIII.2 artificial head, Head Acoustics, Herzogenrath, Germany).

Before beginning the SIFI task, and similarly to the previous tasks, the children completed a ‘practice’ phase during which only visual stimuli were presented across sixteen trials: eight trials with a single flash and 2-2-2-2 trials with double flashes separated by 100, 150, 200 and 300 ms stimulus onset asynchronies (SOA), respectively. Trial order was individually randomized in each block. This phase served multiple purposes: it ensured that participants understood the instructions, and it allowed the experimenter to assist younger children who were unfamiliar with using a keyboard. In such cases, children verbally reported the number of flashes, and the experimenter pressed the corresponding key. The next trial started 1,000 ms after the keypress. If a key was pressed before stimulus presentation, an error message appeared for 2 s, and the trial was re-run. If a key was pressed during stimulus presentation, an error message appeared for 2 s, and the next trial was started. At the end of each block the proportion of correct trials was reported on the screen. Participants progressed to the SIFI task once they achieved at least 80% accuracy. Those who did not reach this threshold repeated the practice phase until they did. All participants successfully completed the practice phase within a maximum of three attempts.

**Figure 3:**
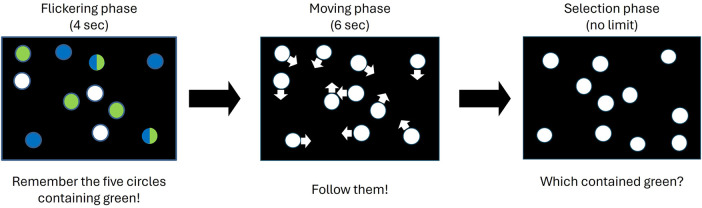
Example trial structure of the MOT task. Participants first memorized the five circles containing green color information. The circles then turned white and moved randomly for 6 s, requiring participants to track the targets among visually identical distractors. Finally, participants selected the circles that had initially contained green. Arrows in the moving phase are shown for illustrative purposes only and were not visible during the task.

The procedure for the testing phase was identical to that of the practice phase, except that participants wore headphones through which auditory stimuli were also presented. Each participant was presented with six blocks, each comprising 64 trials representing six different trial types (Figure 3). Two trial types featured a single event: the presentation of a single flash – ‘F’ trial, or the simultaneous presentation of a flash and a tone –‘FT’ trial); the rest featured double events: two temporally separate flashes (‘F_F’); a simultaneously presented flash and tone followed by a single tone (‘FT_T’) or vice versa (‘T_FT’); a simultaneously presented flash and tone followed by a single flash (‘FT_F’) or vice versa (‘F_FT’); or two simultaneously presented flash and tone (‘FT_FT’) events. Each 64-trial block included 16 fusion-illusory (FT_F and F_FT) and 16 fission-illusory (T_FT and FT_T) trials. Single-flash (F) and one-flash-and-one-tone (FT) trials served as control conditions for the fission illusion, whereas two-flash (F_F) and repeated flash-and-tone (FT_FT) trials served as control conditions for the fusion illusion. In these control trials, the auditory and visual signals were fully congruent (one flash paired with one tone, or two flashes paired with two tones), allowing us to assess participants’ accuracy in discriminating the number of flashes in the absence of conflicting auditory cues. Trials within each 64-trial block were presented in a random order. At the end of each block, the proportion of correct responses, pooled across all non-illusory trials, was reported.

**Figure 4:**
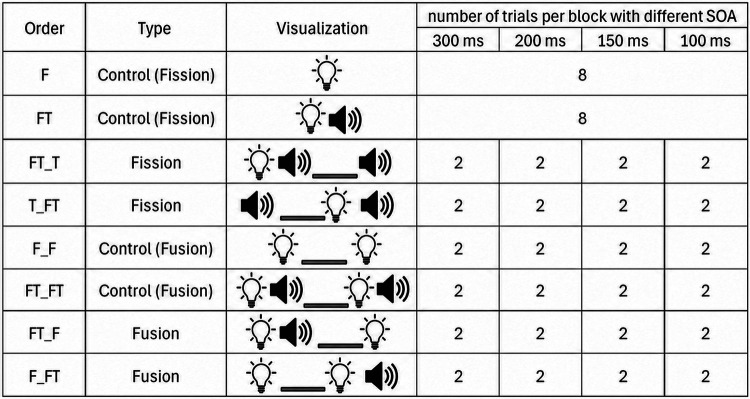
Trial types within a 64-trial block of the sound-induced flash illusion (SIFI) task. F, flash (LED); T, tone; ‘_’ denotes temporal separation between stimuli; SOA, stimulus onset asynchrony. Numbers indicate the number of trials presented for each trial type at each SOA within one block. Control trials contained congruent auditory and visual information, whereas fission and fusion trials contained incongruent audiovisual information designed to elicit illusory perception.

Participants were instructed that they could rest for as long as needed between blocks. SIFI task was administered during both the pre- intervention and post-intervention phases (mean ± SD time elapsed between the two recordings: 314 ± 12.1 days). One control-group participant did not complete the task, and another discontinued after the fourth block during the pre-intervention session. Additionally, data from the last (6th) block of one control participant were lost due to technical issues during the pre-intervention recording (Figure 4).

### 2.3 Data analysis

Statistical analyses were conducted using generalized linear mixed-effect models (IBM SPSS Statistics, version 23) whenever repeated trial-level observations were available, because this approach accounts for the non-independence of observations within participants. Participant ID was included as a random intercept in all GLMMs to control for repeated measures. Fixed effects were selected according to the design of each task and included Group, Session, and task-specific within-participant factors. To address potential developmental confounds, baseline chronological age, expressed in months, was included as a covariate in the primary models. Additional moderation models tested whether age moderated intervention-related change using the Group × Session × Age interaction. Non-significant higher-order interactions were removed stepwise using backward elimination, while lower-order terms involved in significant interactions were retained. Estimated marginal means and pairwise contrasts are reported with standard errors and 95% confidence intervals where applicable. Pairwise comparisons were adjusted using sequential Bonferroni correction, and follow-up tests based on multiple t-tests were corrected using false discovery rate correction, as specified for each analysis. Effect sizes are reported as partial eta-squared values (*η*p^2^), calculated from the model F statistics as *η*p^2^ = (F × df_effect)/[(F × df_effect) + df_error]. Model assumptions were evaluated by inspecting residual distributions and Q–Q plots; where residual distributions showed substantial deviations from normality, appropriate transformations were applied as described below. All statistical tests were two-tailed, and the significance level (α) was set at 0.05.

#### 2.3.1 VSWM task

From the video recordings, response time (i.e., the time elapsed between the appearance of the four options on the screen and the moment the participant pointed to one of them) and accuracy (i.e., whether the participant made a correct choice, coded as 1/0) were extracted for each trial. Because the task involved a speed-accuracy trade-off (i.e., participants were instructed to respond as quickly as possible without sacrificing accuracy), response speed and accuracy were combined into a single performance metric. Specifically, we applied a widely used measure in psychological research, the inverse efficiency score [IES; see (35)]. The IES was calculated by dividing the mean correct response time by the proportion of correct responses for each participant and difficulty level.

The GLMM included Group (CMDT vs. Control), Session (pre-intervention vs. post-intervention), difficulty level (1–3) as fixed effects and Age as covariate. Theoretically relevant two-way interactions were tested initially. Following Q–Q plot analysis of the residuals, which indicated deviations from normality, the square root-transformation of the reciprocal IES values was used as the dependent variable. Non-significant effects were removed stepwise using backward elimination, and sequential Bonferroni adjustment was applied to correct for multiple comparisons.

#### 2.3.2 MOT task

Performance in the multiple object tracking tasks has been extensively investigated from a variety of theoretical perspectives. A commonly used method is the ‘classic’ binomial scoring, in which participants receive a score 1 for correctly identifying a target and 0 otherwise. However, this metric does not account for random guessing, which can artificially inflate accuracy. For example, even when choices are made at random, the probability of correctly selecting the first target (a circle originally contained the color green) is relatively high (5/11 = 0.4545), and this probability increases as incorrect selections reduce the remaining distractors. For instance, after three incorrect choices (i.e., circles originally containing only blue or white), the probability of selecting a correct target rises to 5/8 = 0.625. Therefore, it is reasonable to use a scoring procedure that better accounts for cumulative decision dynamics than simple 1/0 scoring. To eliminate the confounding effect of chance, we applied a method that adjusts for the probability of correct responses: each correctly identified target was awarded 1 minus the actual chance probability of correct choice, whereas each incorrectly identified target was awarded 0 minus the actual chance probability. We have summarized the probability-corrected scores for 1–5 choices per trial to provide a more reliable measure of participant's attentional capabilities. The random intercept generalized linear mixed-effect model included three fixed factors: Group (CMDT vs. Control), Session (pre-intervention vs. post intervention) and Numerical order of trials (from 1 up to maximum 25). All two-way interactions were also included. Non-significant effects were removed stepwise using backward elimination and a sequential Bonferroni adjustment was applied to correct for multiple comparisons. Age was also included as a covariate in the revised primary model, as described above. Importantly, the MOT task not only enables assessment of participants’ general capacity for sustained multifocal attention but also allows examination of their resistance to attentional fatigue across the pre- and post-intervention trials. Although the post-intervention MOT session included 20 test trials whereas the pre-intervention session included 15, only the first 15 post-intervention trials were included in the primary pre–post analyses to ensure an identical number of trials across sessions and to avoid potential confounds related to differences in reliability, learning, or fatigue effects. To examine the effects of the CMDT training on fatigue resistance, we also calculated pre- to post-intervention changes separately for the early (i.e., first five) and late (i.e., last five) test trials of the MOT task. These changes were tested against zero (i.e., no change) using one-sample t-tests with false discovery rate correction [FDRbl adjustment – see (36)].

#### 2.3.3 SIFI task

Each trial was automatically coded as correct (1) or incorrect (0) based on whether the participant correctly identified the target events (i.e., whether one or two flashes were presented). Importantly, two different factors may contribute to incorrect responses in the illusory trials – attentional lapses and reduced sensory precision – whereas errors in the control trials are attributable solely to attentional lapses. Accordingly, to control for the confounding influence of insufficient attention, difference scores were calculated for each participant in each session. These scores were defined as the difference between performance in the illusory trials and the corresponding control trials and served as indices of susceptibility to the fission and fusion illusions. Specifically, susceptibility to the fission illusion was computed as the proportion of incorrect responses in the fission-illusory trials minus the proportion of incorrect responses in the fission-control trials. Susceptibility to the fusion illusion was computed analogously. Participants’ susceptibility to fission and fusion illusions were analyzed separately using random intercept generalized linear mixed-effect models. The models included five fixed factors: Group (CMDT vs. Control), Session (pre-intervention vs. post intervention), SOA (100, 150, 200, 300 ms), Block number (1st – 6th 64-trial blocks) as fixed effects and Age as covariate. All two-way interactions were also included. Non-significant effects were removed stepwise using backward elimination and a sequential Bonferroni adjustment was applied to correct for multiple comparisons. In addition, differences in the susceptibility to fission and fusion illusions between pre- and post-intervention assessments were analyzed separately for each SOA using paired-samples t-tests or Wilcoxon matched-pairs signed-rank tests, depending on the results of Kolmogorov–Smirnov tests of normality. False discovery rate correction (FDRbl adjustment) was applied to account for multiple comparisons.

All statistical tests were two-tailed, and the significance level (*α*) was set at 0.05.

## 3 Results

### 3.1 VSWM task

**Figure 5:**
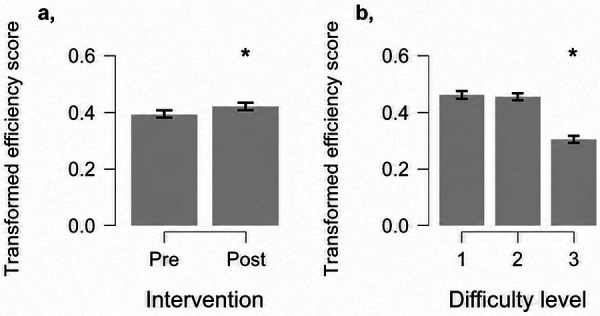
Performance in the visuospatial working memory task. Efficiency scores were calculated as square root-transformed reciprocal inverse efficiency score values and are shown as mean ± SE. Higher values indicate better performance. Panel **(a)** shows performance across pre- and post-intervention sessions, and panel **(b)** shows performance across the three task difficulty levels. Asterisks indicate significant effects (*p < .05).

The GLMM analysis of participants’ performance revealed significant effects of Session (F_1,196_ = 5.251, *p* = 0.023, *η*p^2^ = 0.026), Age (F_1,196_ = 6.785, *p* = 0.010, *η*p^2^ = .034), and Difficulty level (F_2,196_ = 64.693, *p* < .001, *η*p^2^ = .398). Estimated marginal means indicated better overall performance at post-intervention (M = 0.422 ± 0.012, 95% CI [0.399, 0.446]), than at pre-intervention (M = 0.393 ± 0.012, 95% CI [0.369, 0.417]). The pre- to post-intervention contrast was significant (Mdiff = 0.030 ± 0.013, *p* = .023, 95% CI [0.004, 0.055]). For task difficulty, performance was comparable between difficulty levels 1 and 2 (Mdiff = 0.006 ± 0.016, *p* = .686, 95% CI [−0.025, 0.037]), whereas performance was significantly lower at difficulty level 3 than at difficulty level 1 (Mdiff = 0.159 ± 0.016, *p* < .001, 95% CI [0.121, 0.197]), and difficulty level 2 (Mdiff = 0.153 ± 0.016, *p* < .001, 95% CI [0.117, 0.188]). No significant Group effect or Group-related interaction effects were observed. An additional moderation model showed that the Group × Session × Age interaction was not significant (F_1,187_ = 0.557, *p* = .456, *η*p^2^ = .003), suggesting that the absence of a CMDT-specific VSWM effect was not detectably moderated by age (Figure 5).

### 3.2 MOT task

The GLMM analysis revealed a significant main effect of Session (pre-intervention vs. post-intervention; F_1,1015_ = 26.301, *p* < .001, *η*p^2^ = .025), with participants achieving higher probability-corrected scores at post-intervention. A significant main effect of Group (CMDT vs. Control; (F_1,1015_ = 6.134, *p* = .013, *η*p^2^ = .006) was also observed indicating that children in the control group generally performed better in the multiple object tracking task. The analysis further revealed a significant main effect of Age (F_1,1015_ = 10.857, *p* = .001, *η*p^2^ = .011), with older children demonstrating generally better task performance. No significant two-way interaction effects were observed.

**Figure 6:**
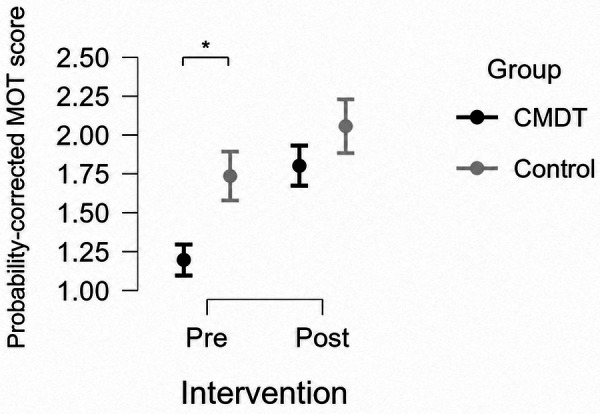
Mean probability-corrected scores (mean ± SE) in the multiple object tracking task for the CMDT and control groups at pre- and post-intervention. Higher values indicate better tracking performance after correction for chance-level responding. Asterisks indicate significant differences (*p < .05).

An additional moderation model showed that the Group × Session × Age interaction was not significant (F_1,970_ = 0.995, *p* = .319, *η*p^2^ = .001), suggesting that the intervention-related change in MOT performance was not detectably moderated by age. However, pairwise contrasts from the age-adjusted model indicated a significant pre- to post-intervention increase in the CMDT group (Mdiff = 0.543 ± 0.188, *p* = .004, 95% CI [0.174, 0.913], whereas the corresponding change in the control group was not significant (Mdiff = 0.325 ± 0.188, *p* = .085, 95% CI [−0.044, 0.695]) (Figure 6).

**Figure 7:**
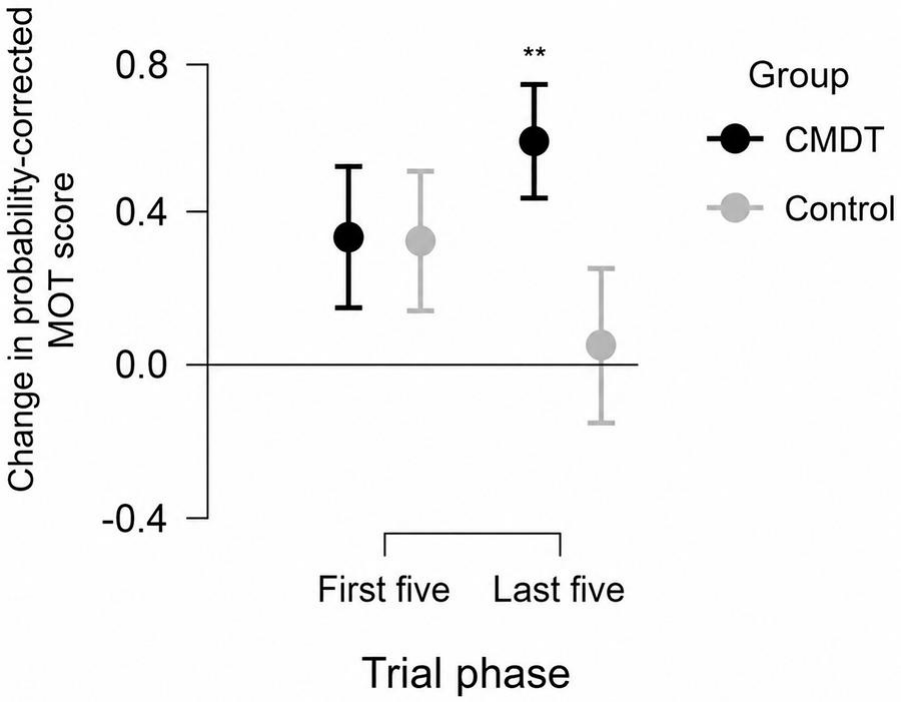
Pre- to post-intervention changes in probability-corrected MOT scores for the early and late phases of the task. Values represent mean ± SE change scores, calculated as post-intervention minus pre-intervention performance. Positive values indicate improvement. The early phase corresponds to the first five test trials, whereas the late phase corresponds to the last five test trials. Significant differences from zero are indicated with asterisks (**p < .001).

The analysis of pre- to post-intervention changes for the early (i.e., first five) and late (i.e., last five) test trials revealed a similar pattern of improvement during the early phase of the MOT task in both the control and CMDT groups (Figure 7). In both groups, the positive change in performance did not reach statistical significance (one sample t-tests: t_84_ = 1.712 for the Control group; t_84_ = 1.752, for the CMDT group; FDR_bl_-adjusted *p* > 0.1 in both cases). However, in the late phase of the MOT task, the CMDT group demonstrated a significant positive change in performance (t_84_ = 3.739, FDR_bl_-adjusted *p* < 0.001), whereas the control group showed no significant improvement (t_84_ = 0.245, FDR_bl_-adjusted *p* > 0.1).

### 3.3 SIFI task

#### 3.3.1 Susceptibility to the fission illusion

**Figure 8:**
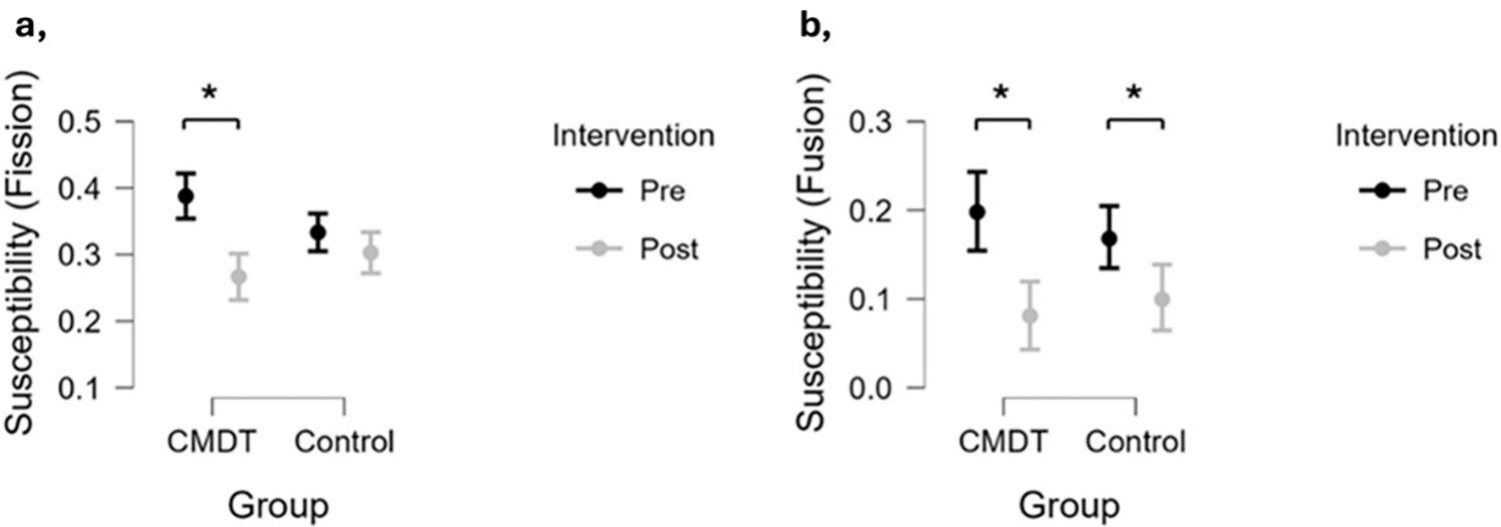
Susceptibility to fission and fusion illusions in the SIFI task. Panel **(a)** shows susceptibility to the fission illusion, and panel **(b)** shows susceptibility to the fusion illusion in the CMDT and control groups at pre- and post-intervention. Values represent mean ± SE. Lower values indicate reduced illusion susceptibility, reflecting greater audiovisual temporal precision. Asterisks indicate significant pre- to post-intervention differences (*p < .05).

The GLMM analysis revealed statistically significant main effect of SOA (F_3,1578_ = 56.198, *p* < 0.001, *η*p^2^ = 0.097), with participants showing progressively greater susceptibility to the fission illusion as a function of stimulus onset asynchrony (100 ms > 150 ms > 200 ms > 300 ms; sequential Bonferroni-adjusted *p* < 0.05 for all pairwise comparisons). The main effects of Session (F_1,1578_ = 26.370, *p* < 0.001, *η*p^2^ = 0.016) and Block number (F_5,1578_ = 10. 502; *p* < 0.001, *η*p^2^ = 0.032) were also significant. The main effects of Group and Age were not significant. However, these effects cannot be interpreted independently due to significant two-way interactions among Group, Session and Block number. The significant Group x Session interaction (Group x Session, F_1,1578_ = 10.219, *p* = 0.001, *η*p^2^ = 0.006) indicates that susceptibility to the fission illusion decreased significantly from pre- to post-intervention in the CMDT group (Bonferroni adjusted *p* < 0.05), whereas no such reduction was observed in the control group (Figure 8A). Pairwise contrasts showed that fission susceptibility did not change significantly in the control group (Mdiff = 0.028 ± 0.021, *p* = 0.179, 95% CI [−0.013, 0.068]), whereas the CMDT group showed a significant pre- to post-intervention reduction (Mdiff = 0.121 ± 0.020, *p* < .001, 95% CI [0.081, 0.160]). The significant Group x Block number interaction (F_5,1578_ = 2. 491, *p* = 0.030, *η*p^2^ = 0.008) indicates that control participants showed significantly greater susceptibility during the first 64-trial block compared to blocks 2–6 (Bonferroni-adjusted *p* < 0.05 for all pairwise comparisons). In contrast, CMDT group demonstrated a more stable performance across blocks 1–6; only 1 vs. 3 and 1 vs. 5 pairwise comparisons indicated significant differences. The main effect of Group and all other interaction effects were non-significant (*p* > 0.05). An additional moderation model showed that the Group × Session × Age interaction was not significant (F_1,1549_ = 0.085, *p* = 0.770, *η*p^2^ < 0.001), suggesting that the CMDT-related reduction in fission susceptibility was not detectably moderated by age.

#### 3.3.2 Susceptibility to the fusion illusion

The GLMM analysis yielded largely similar results. Namely, there was a significant main effect of SOA (F_3,1578_ = 17.180, *p* < 0.001, *η*p^2^ = 0.032) reflecting greater susceptibility to the fusion illusion in the 100 ms condition compared to the 150, 200 and 300 ms SOA conditions (sequential Bonferroni-adjusted *p* < 0.05 for all pairwise comparisons). A significant main effect of Session (F_1,1578_ = 52.418, *p* < 0.001, *η*p^2^ = 0.032) and Age (F_1,1578_ = 6.171, *p* = 0.013, *η*p^2^ = 0.004) was also observed. The significant Group x Session interaction (F_1,1578_ = 4.119, *p* = 0.043, *η*p^2^ = 0.003) indicates that both the control (Mdiff = 0.066 ± 0.018, *p* < .001, 95% CI [0.030, 0.101]) and CMDT groups (Mdiff = 0.117 ± 0.018, *p* < .001, 95% CI [0.083, 0.151]) showed reduced susceptibility to the fusion illusion at post-intervention (Bonferroni-adjusted *p* < 0.05), with greater improvement observed in the CMDT group (Figure 8B). The Group x Block number interaction was also significant (F_5,1578_ = 2.448, *p* = 0.032, *η*p^2^ = 0.008); however, none of the sequential Bonferroni-adjusted pairwise comparisons reached significance (*p* > 0.05) in either group. The main effects of Group and Block number, as well as all other interaction effects, were non-significant (*p* > 0.05). In an additional moderation model, the Group × Session × Age interaction was significant (F_1,1575_ = 8.705, *p* = 0.003, *η*p^2^ = 0.006), suggesting that intervention-related change in fusion susceptibility varied as a function of age.

**Figure 9:**
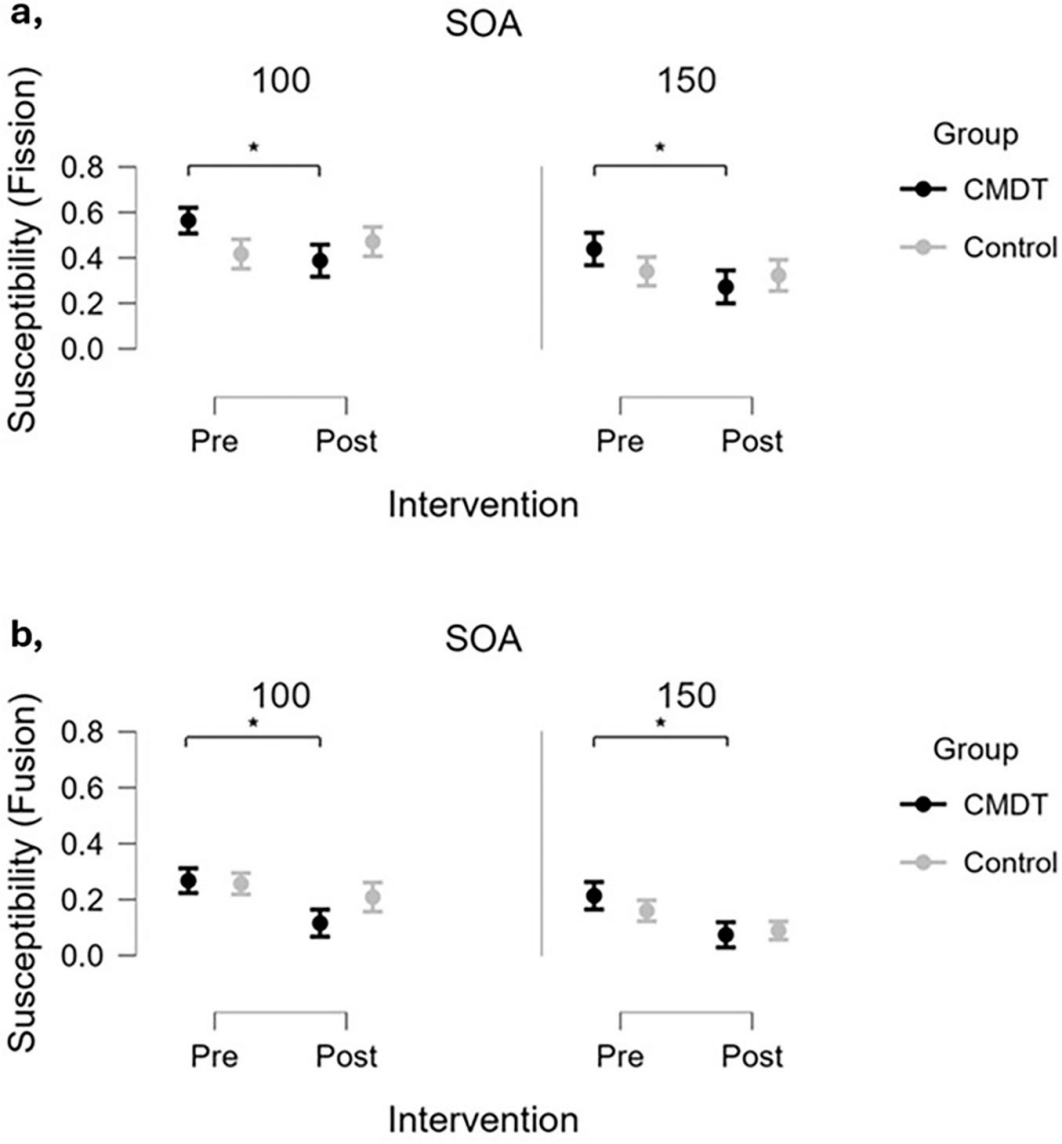
Pre- to post-intervention susceptibility to fission and fusion illusions at the shortest SOA conditions. Panel **(a)** shows susceptibility to the fission illusion, and panel **(b)** shows susceptibility to the fusion illusion at 100 and 150 ms SOAs. Values represent mean ± SE. Lower values indicate reduced illusion susceptibility, reflecting greater audiovisual temporal precision. Asterisks indicate significant pre- to post-intervention differences (* *p* < .05).

Pairwise comparisons of pre- to post-intervention changes in illusion susceptibility revealed distinct SOA-dependent patterns in the Control and CMDT groups. Specifically, the Control group showed no significant pre- to post-intervention changes in susceptibility to either the fission or fusion illusions across any SOA condition. In contrast, the CMDT group demonstrated a significant improvement in sensory precision, reflected by reduced post-intervention susceptibility to both fission and fusion illusions in the shortest SOA conditions (100 or 150 ms; see Figure 9). However, no comparable pre- to post-intervention improvement was observed in the less demanding conditions with longer SOAs (200 and 300 ms; see Supplementary Table S2).

## 4 Discussion

The current study investigated the effects of a 10-month CMDT intervention on U8-U12 soccer players’ visuospatial working memory, multifocal attention, and multisensory integration using three screen-based tasks – VSWM, MOT and SIFI.

### 4.1 VSWM task

The applied visuospatial working memory task required participants to encode a spatial configuration from a horizontal perspective and subsequently identify the correct top-down representation among four rotated alternatives, thereby primarily taxing mental transformation and spatial perspective-taking processes. In addition to the expected effect of the difficulty level of the four response options, our analysis indicated a developmental effect which is consistent with previous findings on the developmental trajectory of visuospatial working memory. Specifically, this cognitive ability follows a prolonged maturation process across childhood and adolescence, with performance typically improving into mid-adolescence (37). Perspective taking, as a core component of visuospatial processing and a central construct assessed in the current study, demonstrates a similarly extended developmental course. Although certain age periods – most notably around ages 8–9 – are characterized by accelerated improvements, maturation in these domains is far from uniform (38), suggesting that chronological age alone does not fully account for the developmental status of these abilities.

However, our analysis did not identify any significant effects associated with CMDT training. The absence of CMDT-specific improvement in VSWM performance may be attributable to a mismatch between the training activities and the assessment task. Although the NORT Complex system presented information at different spatial locations, these locations may not have been sufficiently task-relevant during training to repeatedly engage the spatial transformation and perspective-taking processes measured by the VSWM task. In other words, task-relevant information was primarily encoded in the visual signals displayed on the screen, whereas the spatial location of the displays was likely of little or no relevance during training. It is also possible that the VSWM task was not sensitive enough to detect subtle training-related changes, and that ceiling or floor effects at certain difficulty levels may have limited measurable improvement. Moreover, although visuospatial working memory and perspective-taking abilities continue to develop throughout childhood and adolescence, this maturation is not uniform across the U8-U12 age range which may have further confounded the detection of any training-related effects.

### 4.2 MOT task

The analysis indicated a pattern of CMDT-related improvement in the multiple object tracking task. Specifically, although the Group × Session interaction did not reach statistical significance after controlling for age, pairwise contrasts revealed greater pre- to post-intervention improvement in the CMDT group, particularly in the later phase of the task, where the control group showed little change. Overall, these changes suggest a positive effect of CMDT training on children's resistance to attentional fatigue.

This task places substantial demands on sustained attentional allocation, dynamic updating, and resistance to interference – processes that closely resemble the perceptual-attentional demands embedded in the NORT Complex smart coaching training sessions. Continuous visual scanning and the sustained allocation of attention across multiple moving elements closely align with the attentional control demands of CMDT training sessions. This correspondence may explain why improvements were observed in MOT but not in VSWM performance, in the CMDT group.

The ability to efficiently distribute visual attention across multiple potential targets has been identified as a key factor distinguishing high- from mid-level soccer players, even before motor execution in passing situations (39). These differences may partly reflect the sustained cognitive demands imposed by CMDT training, which requires continuous allocation of attention to task-relevant stimuli appearing at spatially distinct locations across the playing field. By placing players in an active information-seeking role, rather than that of a passive listener, such training may promote more effective visual scanning and attentional control strategies. This effect is particularly relevant under elevated physical load, as increased physical demands can impair attentional and perceptual processing (40). In this context, CMDT training may therefore help mitigate load-related declines in visual attention by reinforcing the efficiency of attentional resource allocation in complex, dynamic environments. This interpretation is supported by the performance pattern observed in the MOT task. Both groups showed comparable, statistically non-significant improvements relative to the pre-intervention measurement during the initial five trials; however, a significant increase in probability scores in the final five trials was observed only in the CMDT intervention group, whereas the control group showed no improvement. Previous studies using CMDT-based interventions have also reported positive effects on attentional performance in volleyball (41) and basketball (42). Lucia and colleagues (42) further suggested that such benefits may reflect neuroplastic adaptations, evidenced by enhanced anticipatory processing in event-related potentials among adolescents. These changes were associated with more efficient anticipatory activity in prefrontal cortical regions involved in attentional control and prediction. Given that participants in the present study were younger than those examined by Lucia et al. (42), it is plausible that similar neuroplastic mechanisms contributed to the training-related improvements observed in the CMDT intervention group. Fleddermann and colleagues (43) implemented an 8-week intervention where elite volleyball players performed a 3D-MOT task simultaneously with either sport-specific or general motor drills. While the program successfully improved task-specific tracking performance and near-transfer outcomes such as processing speed, it failed to produce significant far-transfer effects in a game-like blocking test. The authors attributed this limitation to the low correspondence between the abstract training stimuli and the complex visual-tactical demands inherent in real-game situations. Furthermore, persistent dual-task costs observed during the blocking test suggested that off-court, generic training does not readily integrate into sport-specific motor patterns. These results suggest that, for effective transfer to occur, cognitive-motor dual-task training should be implemented directly within the actual sport environment to better align with its situational demands. Accordingly, rather than relying on isolated screen-based tasks, we propose that distributing training devices across the playing field – as in the present study – and imposing continuous scanning demands by dynamically altering both the location and meaning of stimuli during real training situations may directly enhance multifocal attention.

### 4.3 SIFI task

Significant effects were also identified in relation to multisensory integration or sensory precision processes. Across participants, susceptibility to the fission illusion was substantially greater – nearly twice as high – than susceptibility to the fusion illusion, replicating prior findings suggesting auditory dominance in the processing of temporally defined events within this age range (44). Importantly, children in the CMDT group demonstrated reduced susceptibility to both illusion types following the intervention, particularly at the shortest (100–150 ms) SOAs, where perceptual ambiguity is highest. However, the pattern differed between the two illusion types. For fission susceptibility, the CMDT group showed a significant pre- to post-intervention reduction, whereas no comparable reduction was observed in the control group. For fusion susceptibility, both groups showed reduced susceptibility at post-intervention, but the reduction was larger in the CMDT group. This pattern suggests that the improvements cannot be attributed solely to developmental maturation during this period, and the CMDT intervention may have contributed to improved audiovisual temporal precision, although the effects were not identical for fission and fusion illusions.

This finding contributes to ongoing debates within multisensory integration research [see (45) for a review] concerning the timeline of maturation for sensory precision and cross-modal binding processes. The additional moderation analyses further indicated that these effects should be interpreted in relation to age. For fission susceptibility, the CMDT-related reduction was not detectably moderated by age. For fusion susceptibility, however, the Group × Session × Age interaction was significant, suggesting that intervention-related change may have varied across the U8–U12 age range. Given the small effect size, this age-dependent moderation should be treated as exploratory and interpreted cautiously. Additionally, participants assigned to the control group showed evidence of a within-task sensory precision effect: their tendency to commit fission illusion errors decreased significantly after the first 64-trial block. In contrast, CMDT participants exhibited a more stable perceptual processing profile across blocks. This pattern may suggest that the CMDT intervention has the potential to reduce short-term fluctuations in illusion susceptibility. While the relationship between CMDT training using the NORT Complex smart coaching system and multifocal attention appears relatively straightforward, their effects on sensory processing are less easily interpreted. A recent review by Hirst and colleagues (46) summarized the past twenty years of research on the sound-induced flash illusion (SIFI), noting that most empirical studies have focused on the fission illusion, whereas the fusion phenomenon has remained largely unexplored. The authors concluded that early interactions within the primary sensory cortices primarily contribute to the fission illusion and that fission and fusion likely rely on distinct neural mechanisms. This is supported by the finding that children had similar performance to adults for fusion, but not for fission (47). Performance in the SIFI task has also been shown to be influenced by long-term regular exercise (48) with improvements potentially underpinned by increased cerebral activity (49). Although these findings are based on studies with young adults, comparable effects have been reported following an acute bout of physical exercise in children aged 6–8 years (50). While these results may partly explain the improvements observed between the first and second measurements as a general effect of regular physical activity (i.e., weekly training sessions), the difference between the CMDT and control groups suggests that exercise alone cannot fully account for the observed pattern of results.

Reviews on the development of sensory dominance and multisensory integration indicate that visual dominance typically emerges around 9–10 years of age and becomes consolidated between 11 and 12 years (44). However, optimal multisensory integration may continue to develop into later adolescence (45). Considering these developmental and exercise-related findings alongside the results of the present study, it may be hypothesized that cognitively demanding physical activity involving rapid decision-making – such as the CMDT intervention applied here – can facilitate more efficient sensory processing and multisensory integration. Training contexts that require continuous monitoring of multiple stimuli under time pressure may engage attentional control networks and promote adaptive sensory reweighting, whereby task-relevant inputs – predominantly visual in game situations – gain priority over less informative cues. Repeated exposure to such demands may therefore strengthen the flexibility and precision of cross-modal integration processes. This mechanism may be particularly relevant during the development of younger athletes. In traditional training environments, players may rely more heavily on auditory cues, such as the coach's verbal instructions, potentially reinforcing a sensory hierarchy that differs from the demands of competitive play. In contrast, cognitively enriched, game-like training environments may recalibrate attentional weighting and sensory prioritization, fostering perceptual processing strategies that more closely align with the perceptual demands of match situations.

### 4.4 Practical recommendations for coaches

The present findings suggest that cognitively enriched, field-based training may be a useful complement to traditional soccer practice in youth development programs. From a practical perspective, coaches may consider integrating cognitive demands directly into regular technical and tactical drills, rather than treating cognitive training as a separate, screen-based activity. Exercises may be designed so that players must continuously scan the environment, respond to spatially distributed visual cues, and update their actions while dribbling, passing, positioning, or cooperating with teammates. Such training should be introduced progressively and remain age appropriate. For younger players, cognitive load can be increased gradually by manipulating the number of relevant cues, the speed of cue presentation, the number of possible response rules, or the need to divide attention between the ball, teammates, opponents, and external signals. Importantly, cognitively enriched drills should not replace the development of fundamental technical and tactical skills; rather, they should be embedded within these activities to better approximate the demands of real game situations. The present results suggest that this approach may be particularly relevant for perceptual-attentional processes, such as multifocal attention and rapid multisensory processing. In contrast, improvements in visuospatial working memory may require training protocols in which spatial information must be explicitly maintained, manipulated, or transformed. Coaches aiming to target specific cognitive functions should therefore carefully align the cognitive demands of the drill with the intended developmental outcome.

### 4.5 Limitations and future directions

Although children were evenly assigned to the CMDT training and the control groups based on their biological- and sporting age as well as their coaches’ evaluation of player ability, a limitation of the present study is that the two groups were not balanced with respect to their baseline (i.e., pre-intervention) measures of cognitive performance. Baseline cognitive differences may therefore have partially contributed to group-related changes, thereby limiting the strength of causal interpretations. This concern is particularly relevant for the multiple object tracking task, in which the control group demonstrated significantly better performance than the CMDT group during the pre-intervention assessment. A further limitation is that the present design cannot fully separate the effects of CMDT-specific training content from the effects of training volume (the total training exposure was 14.1% higher in the CMDT group than in the control group). This imbalance reflects the ecological nature of the intervention: the study was conducted within an active talent development program, and it was not feasible to reduce or replace players’ regular soccer training to achieve perfectly matched training loads. Although the effects of training volume cannot be ruled out, the overall pattern of results does not suggest a simple dose-response relationship between total training exposure and cognitive outcomes Specifically, the strongest intervention-related effects were observed for SIFI outcomes, whereas the MOT findings were more attenuated after controlling for age and no CMDT-specific effect was observed for VSWM. Future studies should therefore include an active control group matched for total training duration to isolate the specific contribution of CMDT content more precisely.

While the present study adopted a longitudinal perspective – which remains relatively rare in research examining the effects of CMDT training on athletes’ performance – future studies should extend this approach by investigating the long-term effects of CMDT interventions across different age groups and levels of sport expertise. Future research should also explore whether cognitively enriched training protocols can be adapted to target different components of executive functioning, including visuospatial working memory. While the current study integrated the NORT Complex smart coaching system into a traditional coaching framework to highlight the technology's inherent benefits, these findings point to a broader potential for athlete development. Building on the present findings regarding on-field device placement and the continuous scanning demands it imposes, future research should advance toward the development of dedicated training protocols grounded in CMDT principles. By leveraging the system's capacity to precisely and directly target executive functions, such novel methodological approaches could potentially yield substantially greater developmental gains than traditional training models. In addition, future studies should assess whether CMDT interventions offer unique advantages over traditional training in refining sensory precision. Particular emphasis should be placed on identifying the distinct roles these training types play in modulating attentional control and sensory reweighting – key mechanisms underlying the efficient multisensory integration and the narrowing of the temporal binding window. Investigating athletes ranging from novices to elite performers would enable a more nuanced understanding of how developmental stage and training background interact with training-related cognitive adaptations and would help clarify the durability and generalizability of CMDT-induced effects.

## 5 Conclusions

In conclusion, integrating cognitive and motor demands within athletic training is essential for bridging the gap between laboratory-based cognitive gains and real-game performance. This study suggests that longitudinal cognitive-motor dual-task training may support perceptual-attentional and multisensory processes in juvenile soccer players, with the most robust effects observed for multisensory precision. Our findings add a further piece of evidence to the increasingly accepted notion that systematically incorporating additional cognitive challenges into the training of sport-specific motor skills may represent an efficient methodological approach to athlete development. While the absence of transfer to visuospatial working memory suggests that training effects may be domain-specific or dependent on the nature of the exercises, increasing the cognitive complexity of sport-specific environments remains a timely and relevant strategy for athlete development. Establishing evidence-informed CMDT guidelines across different developmental stages may help ensure that the neural functions governing athletic behavior are optimally supported and effectively generalized.

## Data Availability

The raw data supporting the conclusions of this article will be made available by the authors, without undue reservation.

## Footnotes

^1^NORT Complex. http://nortcomplex.com/en/kezdolap-2eng/

^2^Csound [Computer software]. http://csounds.com
