# Supplementary material for: Cognitive-motor dual-task training differentially affects visual working memory, multifocal attention, and multisensory integration in youth soccer players
Source: Front Sports Act Living. 2026 Jul 22;8:1863511. doi: 10.3389/fspor.2026.1863511 (PMC13438399; doi:10.3389/fspor.2026.1863511)
Supplement: Supplementary file 1 [file Table1.docx]

**Supplementary materials**

|  | |  | **correct choices (%)** | | **RT - correct choices (sec)** | | **RT - incorrect choices (sec)** | |
| --- | --- | --- | --- | --- | --- | --- | --- | --- |
|  | |  | pre-intervention | post-intervention | pre-intervention | post-intervention | pre-intervention | post-intervention |
|  | group | difficulty level | mean±SE | | mean±SE | | mean±SE | |
|  | Control | 1 | 0.79±0.05 | 0.81±0.04 | 4.39±0.44 | 4.0±0.43 | 5.25±0.98 | 6.06±0.86 |
|  |  | 2 | 0.76±0.05 | 0.87±0.04 | 3.86±0.27 | 3.65±0.28 | 5.61±0.62 | 4.46±0.62 |
|  |  | 3 | 0.38±0.05 | 0.52±0.05 | 5.24±0.56 | 5.03±0.55 | 5.70±0.92 | 4.85±0.51 |
|  | CMDT | 1 | 0.79±0.04 | 0.75±0.05 | 4.24±0.58 | 3.77±0.36 | 5.55±1.07 | 4.48±0.54 |
|  |  | 2 | 0.62±0.07 | 0.74±0.05 | 4.17±0.48 | 3.51±0.24 | 4.24±0.37 | 4.94±0.54 |
|  |  | 3 | 0.33±0.05 | 0.38±0.04 | 4.79±0.81 | 5.03±0.69 | 4.81±0.56 | 6.12±0.74 |

**Table S1.** Mean (± SE) values of the proportion of correct choices and response times (RTs) for correct- and incorrect trials in the visual working memory (VSWM) task across different phases (pre- vs post intervention) and trial difficulty levels (1-3).

| **Susceptibility to fission illusion** | | | | | |
| --- | --- | --- | --- | --- | --- |
|  |  | pre-intervention | post-intervention |  |  |
|  | SOA (ms) | mean±SE | | statistics | p |
| Control group | 100 | 0.417±0.065 | 0.471±0.065 | t(15)=0.743 | NS |
|  | 150 | 0.340±0.063 | 0.323±0.068 | t(15)=0.229 | NS |
|  | 200 | 0.339±0.055 | 0.224±0.046 | t(15)=1.992 | NS |
|  | 300 | 0.237±0.034 | 0.193±0.044 | t(15)=1.048 | NS |
| CMDT intervention group | 100 | 0.564±0.057 | 0.387±0.071 | t(16)=2.975 | **<.05** |
|  | 150 | 0.439±0.045 | 0.272±0.071 | t(16)=2.823 | **<.05** |
|  | 200 | 0.328±0.063 | 0.228±0.070 | n=17, Z=-1,371 | NS |
|  | 300 | 0.221±0.055 | 0.179±0.060 | n=17, Z=-0,943 | NS |
| **Susceptibility to fusion illusion** | | | | | |
|  |  | pre-intervention | post-intervention |  |  |
|  | SOA (ms) | mean±SE | | statistics | p |
| Control group | 100 | 0.256±0.038 | 0.208±0.052 | n=16, Z=-0,227 | NS |
|  | 150 | 0.159±0.037 | 0.089±0.032 | t(15)=2.236 | NS |
|  | 200 | 0.129±0.030 | 0.044±0.020 | n=16, Z=-1,993 | NS |
|  | 300 | 0.128±0.026 | 0.057±0.027 | n=16, Z=-2,099 | NS |
| CMDT intervention group | 100 | 0.267±0.044 | 0.115±0.048 | n=17, Z=-3,040 | **<.05** |
|  | 150 | 0.213±0.072 | 0.074±0.049 | n=17, Z=-3,427 | **<.05** |
|  | 200 | 0.162±0.051 | 0.088±0.033 | n=17, Z=-1,382 | NS |
|  | 300 | 0.150±0.038 | 0.047±0.029 | t(16)=3.068 | **<.05** |

**Table S2.** Mean (± SE) values and pairwise comparisons of illusion susceptibility across stimulus onset asynchrony (SOA) conditions in the sound-induced flash illusion (SIFI) task. To account for multiple comparisons, p < .05 indicates statistical significance at the α = .05 level following false discovery rate correction (FDRbl adjustment).
